# The relationship between the intensity of *Gasterophilus intestinalis* larvae infection and the serum and salivary humoral immune response in horses

**DOI:** 10.1038/s41598-022-21482-z

**Published:** 2022-10-20

**Authors:** Monika Pawlas-Opiela, Paulina Jawor, Józef Galli, Agnieszka Zak-Bochenek, Michał Gorczykowski, Joanna Galli, Zenon Sołtysiak, Tadeusz Stefaniak

**Affiliations:** 1 Department of Immunology, Pathophysiology and Veterinary Preventive, Faculty of Veterinary Medicine, Wrocław University of Environmental and Life Sciences, C. Norwida 31, 50-375 Wrocław, Poland; 2 Department of Internal Diseases and Clinic of Diseases of Horses, Dogs and Cats, Faculty of Veterinary Medicine, Wrocław University of Environmental and Life Sciences, Pl. Grunwaldzki 47, 50-366 Wrocław, Poland; 3 Present Address: Department of Diagnostics and Clinical Sciences, Institute of Veterinary Medicine, Nicolaus Copernicus University, 87-100 Toruń, Poland

**Keywords:** Immunology, Gastroenterology

## Abstract

Infection with *Gasterophilus intestinalis* (botfly) larvae often occurs in horses. The aim of the study was to isolate the larvae of *G. intestinalis* and evaluate the serum and salivary humoral immune response using self-developed ELISA in *G. intestinalis* infected horses. Blood serum or saliva samples were taken from 125 infected horses and 54 uninfected slaughtered horses. The antigens from *G. intestinalis* larvae were used for development of ELISA in order to evaluate the intensity of *G. intestinalis* IgG, IgM, and IgA antibody reactivity in the serum or saliva of naturally infected horses and horses without larvae in the gastrointestinal tract (control group). Serum antibodies against second and third larvae’s stadium antigens reacted significantly more intensively in infected than in healthy horses in IgG (*p* ≤ 0.001; *p* ≤ 0.05, respectively) and IgA (*p* ≤ 0.05;*p* ≤ 0.001, respectively) classes. Salivary IgG and IgA specific’s antibody reactivity was significantly higher in horses with moderate (*p* ≤ 0.01) and severe infection (*p* ≤ 0.001) compared to the healthy horses. The determination of the *G. intestinalis* IgG and IgA antibody activity in saliva and serum may be used for detecting horses moderately and severely infested with larvae.

## Introduction

Botflies are parasites that, in their larvae instar, parasitize in Equidae, almost all over the world^1^. The most popular species in Central Europe comprise *Gasterophilus intestinalis* (> 90%) and *Gasterophilus nasalis* (< 10%)^2^. Infections spread mainly in large horse groups, and diagnostic difficulties make it necessary to introduce routine deworming without making a diagnosis^1^. Gasterophilosis may be associated with gastrointestinal complications (gastrointestinal ulcerations, colics, rectal prolapse, diarrhoea, and digestive disorders), due to L2 and L3 activity^3,4^ There is no confirmed relationship between the presence of botfly larvae and stomach ulceration in rural horses, however the third larvae instar (L3) may attach non-glandular part of the stomach mucosa causing inflammation^2,3,6^. Clinical signs of infection may alsobe connected with the migration of the first larvae instar (L1) and the second larvae instar (L2) within the oral cavity, where profound damage of the epithelium and mucosa of the cavity, including the tongue and gums may occur^5,7^. The observable signs, which are significant when a horse is designated for sport use, comprise excessive salivation, tenderness of the tongue, head shaking, difficulties in swallowing and pain^5,7^. The diagnosis of botfly infestation is a significant clinical challenge. The L1 invasion may also be found during oral inspection, where the larvae locate in the diastemas between premolar and molar teeth or on the basis of mucosal damage^7^. Griss and Simhofer in endoscopic examinations of the oral cavities of the horses manifesting pain upon feeding, found the presence of larvae instar L2 located in the oral cavity^5^. L2 and L3 may be detected upon endoscopic inspection^8^. The subject literature describes serological tests which allow for detecting, in the equine blood serum, IgG antibodies against *G. intestinalis* and *G. nasalis* antigens, whose reactivity varied with the season, which could be due to the immunogenicity of individual larval instars^9^. A quick and early diagnosis has great significance on account of the risk of inducing, by developing larvae, clinical symptoms related to inflammation and pain in the gastrointestinal system; in sport horses, this can significantly affect the locomotory system and exercise capacity^3,4^. The objective of this paper was to isolate the antigens from *G. intestinalis* larvae and then to develop the conditions for the ELISA to detect the antibodies against the isolated antigens of instars L2 and L3 *G. intestinalis* in IgG, IgM and IgA classes in blood serum and saliva of horses with various intensity of infection.

## Results

**Figure 1:**
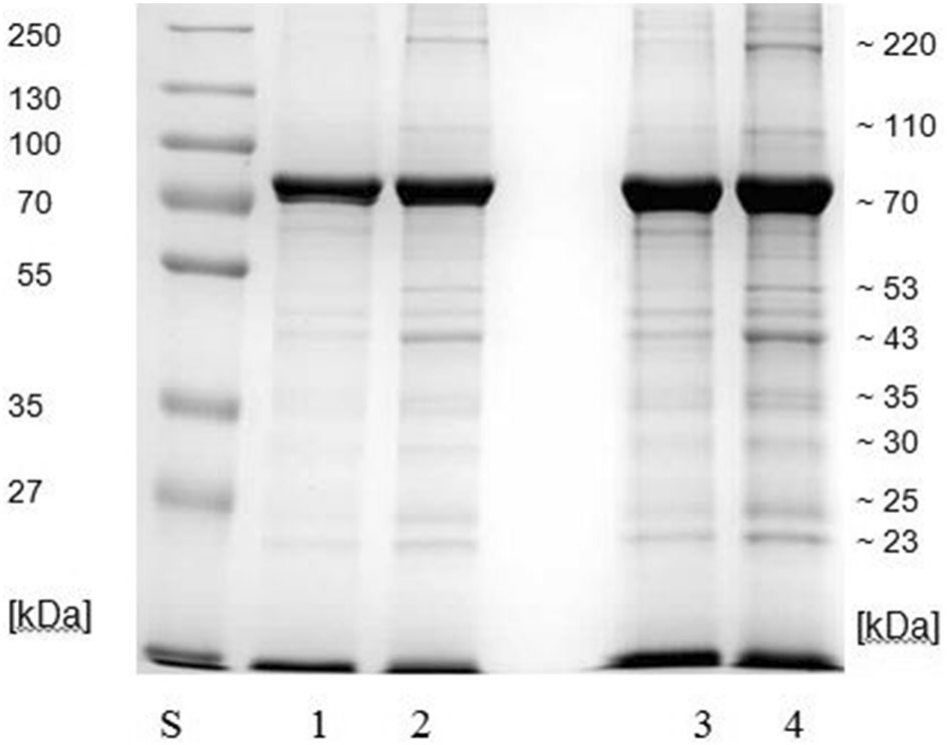
Electrophoretic separation of the proteins of *G. intestinalis* antigen with SDS-PAGE method (all antigens presented on one blot). S—(molecular weight standard). 1—antigen obtained from the anterior part of the 3 L2 *G. intestinalis* larva. 2—antigen obtained from the entire L2 *G. intestinalis* larva. 3—antigen obtained from the anterior part of the 3 L3 *G. intestinalis* larva. 4—antigen obtained from the entire L3 *G. intestinalis* larva. (The raw version of blot is available in the Supplementary materials section).

As a result of the 1st step of presented studies, crude antigen from L3 (from 1 larva) was obtained with protein concentration of 3.2 mg/ml; as well as antigen from the anterior part of the L3 larva body with protein concentration of 1.8 mg/ml; antigen from L2 (from 1 larva) with protein concentration of 1.24 mg/ml and antigen from the anterior part of the L3 body with protein concentration of 0.6 mg/ml. The electrophoretic patterns of the proteins of the *G. intestinalis* L2 and L3 antigen, both from the anterior parts of the larva and the entire bodies, respectively were comparable and presented similar protein fractions with the same specific molecular weights ranging from ~ 23 to ~ 250 k-Da (Fig. 1). The largest fraction consisted the proteins with the molecular weight of ~ 70 k-Da. Remaining protein bands of slightly stronger staining than other fractions had the molecular weight of ~ 43 ~ 220 and ~ 23 k-Da. They manifested significantly larger density in the proteins isolated from the entire larvae than only from their rostral part. The protein pattern of the antigen with is presented on Fig. 1.

The reactivity of antibodies (IgG, IgM and IgA classes) against *G. intestinalis* in the serum from horses from 2nd step of the study was presented in Table 1. The horse serum revealed the presence of IgG, IgM and IgA antibodies against *G. intestinalis* antigens isolated from both developmental instars, i.e. L2 and L3. The reactivity of the antibodies in IgG class was significantly higher in the infected individuals in comparison with healthy ones, both against L2 (*p* ≤ 0.001) and L3 (*p* ≤ 0.05). The reactivity of specific antibodies in the IgA class was significantly higher in infected individuals in comparison with healthy ones, both against L2 (*p* ≤ 0.05) and L3 (*p* ≤ 0.001) antigens. No difference was found between the groups in serum IgM class antibody activity.

**Table 1:** Results of the 2nd Stage of the study: Mean reactivity (SD) of serum antibodies in classes IgG, IgM and IgA in the horses free from invasion and infected ones (> 50 larvae) with antigens isolated from L2 and L3 *G. intestinalis* in the ELISA test (A = 492 nm).

Ig class	The antigen used	Uninfected (n = 30) mean (SD)	Infected (n = 30) mean (SD)
IgM	L2	0.543 (0.13)	0.558 (0.14)
	L3	0.541 (0.13)	0.560 (0.10)
IgG	L2	0.184 (0.08)	0.293*** (0.13)
	L3	0.214 (0.10)	0.275* (0.11)
IgA	L2	0.124 (0.02)	0.154* (0.06)
	L3	0.113 (0.03)	0.134*** (0.03)

Legend: L2—larval stage II, L3—larval stage III, statistical significance: *p ≤ 0.05, **p ≤ 0.01, ***p ≤ 0.001, in comparison with the horses free from invasion.

The reactivity of the saliva IgG, IgM and IgA antibody against *G. intestinalis* in respective groups from 3rd step of the study (I, II, III and IV) was presented in Table 2. ELISA performed on the horse saliva revealed a positive reaction in all the examined horse groups. In group III (51–150 larvae) the antibodies reactivity was significantly higher in IgG and IgA classes (*p* ≤ 0.01 and *p* ≤ 0.001) respectively in comparison with group I (in which no larvae were found). In group IV (> 200 larvae) the saliva Ab reactivity was significantly higher in all the examined classes, i.e. IgG (*p* ≤ 0.001), IgM (*p* ≤ 0.05) and IgA (*p* ≤ 0.001) in comparison with group I.

**Table 2:** Results of the 3rd Stage of the study: Mean reactivity (SD) of antibodies in saliva in IgG, IgM and IgA classes in horses free from invasion and manifesting diverse intensity of invasion u with antigens L3 *G. intestinalis* in the ELISA test (A = 492 nm).

Ig class	Group I Uninfected n = 24 mean (SD)	Group II (10–50 larvae) n = 15 mean (SD)	Group III (51–150 larvae) n = 20 mean (SD)	Group IV (> 200 larvae) n = 20 mean (SD)
IgM	0.225 (0.07)	0.292 (0.11)	0.294 (0.07)	0.354* (0.11)
IgG	0.213 (0.07)	0.260 (0.06)	0.280** (0.09)	0.393*** (0.11)
IgA	0.387 (0.14)	0.410 (0.14)	0.585** (0.15)	0.919*** (0.25)

Legend: statistical significance: *p ≤ 0.05, **p ≤ 0.01, ***p ≤ 0.001, in comparison with Group I.

## Discussion

The study of the immune response of animals with gastric myiasis caused by *Hypoderma bovis, Oestrus ovis, Gasterophilus sp.* show that larvae antigens that induce an immune response in the host are not connected with the surface structure of their bodies, but, instead, mostly with the gastric enzymes of the salivary gland excretion^9–15^. Based on the observations of the above authors, and also on the fact that botfly larvae attach to the mucosal membranes of the host with their mouthpart, in our study it was decided to isolate the antigens from the anterior part of the body of the larvae and also from the entire larvae (mixture). The electrophoretic separation of the proteins obtained, both, from the anterior part of the body of the L2 and L3 and whole bodies L2 and L3, showed a similar results to the study of Roelstra et al.^16^. In the quoted study, thick bands were detected as well as at the level of molecular weight of approximately 17 kDa, but in that study gels were stained with silver which is much more sensitive than Coomassie blue^16^. Some of the differences between this and our study may be due to different procedures in isolating antigens from the larvae.

In the current study there was no difference in serum IgM antibody reactivity between infected horses and those which had no larvae detected in the stomach. The lack of any difference between the control group and the group infected for the IgM call may result from an earlier contact with the parasite. Viviers et al. used the test of passive hemagglutination in the diagnostics of botfly infection in horses^17^. They observed that in the serum of the foals infected experimentally with the larvae of *G. intestinalis,* hemagglutinating antibodies were present already in the third week after infection. However, in the case of *G. nasalis* infection, hemagglutinating antibodies were found in the foal serum 8 weeks after infection^17^. A slow decrease in the level of antibodies was observed by the authors within a period of up to 12 weeks after infection^17^. We do not know how long the horses had been infected with *G. intestinalis* before sampling, but serum IgG and IgA class were significantly higher compared to uninfected horses. The study carried out in horses by Sanchez-Andrade et al. showed some seasonal changeability of the serum IgG antibody titre against secretory antigens of L2 *G. intestinalis* (GphilL2ES) and L2 *G. nasalis* (GphnL2ES)^9^. In winter, an increase of reaction intensity was observed, which was most probably related to the presence of L2 and L3 in the gastrointestinal tract^9^. However, that study did not relate seroprevalence to the actual degree of parasite invasion in the stomach and duodenum, as only an observation of *Gasterophilus* eggs attached to the hair and/or 3rd instar larvae in faeces^9^. On the other hand, in the current study, the effect of seasonality was not determined, so it is not known to what extent there may be variation in the level of antibodies in horses throughout the year. The use of measurements of the specific antibodies with the ELISA is common in veterinary medicine. Sheep infection with *Oestrus ovis* larvae has become the subject of many experiments^14,18^. Tabouret et al. used the ELISA for the diagnostics of *Oestrus ovis* infection in sheep and showed that the test sensitivity and specificity varied depending on the season of the year and the phase of the animal's infection^14^. The results of the studies performed by Suárez et al. showed that the presence of specific IgG serum antibodies is not always indicative of the ongoing invasion, as it may also be the result of an earlier contact of an animal with a parasite^18^. Certainly, the intensity of the reaction and the isotype of the *G. intestinalis* antibodies depend on the moment of infection, the intensity of invasion and the individual properties of the horse (low–high responders), the type of anthelmintics used, and earlier contacts of the horses with the parasite^9^.

The results of tests of saliva samples in which the ELISA test was used to measure the level of IgA, IgM and IgG antibodies specific for *G. intestinalis* L3 are promising. The highest intensity of reaction to the examined parasite antigens was observed in the IgA class which is the main immunoglobulin class in mucosal secretions^19^. Significantly higher reactivity of both IgA and IgG antibodies in horse saliva was observed in horses in whose stomachs and duodenums more than 50 larvae were found; whilst in the base of IgM antibodies, the same level of reactivity was seen only in intensively infected horses (> 200 larvae). According to the literature, secretory IgA plays also an important role in fighting parasitic infections of the gastrointestinal tract^20^. In sheep, it has been shown that salivary IgA directed against CarLA (the antigen present on the surface of 3 instar larvae of intestinal nematodes) connected with surface antigens, prevents the larvae settlement in the preferred places of intestinal epithelium^20^. Significantly higher activity of IgA antibody was detected in the saliva of the horses with more than 50 *G. intestinalis* larvae found in their stomachs and also the lack of significant differences in the intensity of reaction of the IgA antibody against *G. intestinalis* in the saliva, observed between the horses in whose case low infection intensity was observed (10–50 larvae), and the horses free from any invasion, seem to be interesting from the point of view of diagnostic usefulness of this method in detecting high burden horses. From a diagnostic point of view, detecting specific IgA antibodies in saliva is used in human medicine for diagnosing protozoa invasion *Naegleria fowleri*^21^. So far, in the diagnostics of parasitic diseases of horses, serological tests to detect serum and saliva antibody may be used for the assessment of tapeworm infections^22^. Both the tests based on processing serum and saliva are characterized by high sensitivity (85% and 83%) and specificity (78% and 85%, respectively)^22^. The commercially available test measuring the intensity of tapeworm infection, on the basis of saliva examination, is not only a non-invasive method, but also an easily applicable method since, saliva may be collected by the horse owner. In the case of the diagnostics of *G. intestinalis* stool analysis seems to have a low diagnostic value^22^. Similarly as in the case of tapeworm diagnostics, the proposed test, based on detecting specific IgA in horse saliva as an indicator of *G. intestinalis* infections, seems to be a sensible completion of the diagnostic and decision-making process concerning deworming the herd. The obtained results illustrate the relationship between the intensity of horse infection with *G. intestinalis* larvae and the antibody activity in the horse serum and saliva (in particular IgA and IgG in saliva).We are aware of some limitations of this study. Some further studies of myasis in horses should definitely identify whether the evaluation of antibody reaction intensity in the horse saliva has any reference to the presence of the L1 and L2 in the oral cavity, which might complete the diagnostic methods applied when atypical pain symptoms of the dental system occur. Despite the fact that only horses with *G. intestinalis* were selected for the study, it cannot be ruled out that there is some cross-reactivity with other parasites will be present. Although more work is needed in order to ameliorate the limitations of this study, the Authors are confident that the salivary approach will give easy and fast prediction of Gasterophilus presence in the horses, and this seems to be a useful non-invasive parameter of intensity of infection.

## Material and methods

## Study design

The study was performed in three steps:

Step 1st: isolation of the *G. intestinalis* larvae from the gastrointestinal tract of 10 naturally infected horses upon slaughter. The larvae, isolated from the stomach and duodenum were placed in 0.9% saline solution. The development instar of the larvae was determined, with differentiation into immature – L2, and mature – L3 [,23,24]. After a morphological assessment, the larvae were placed in 10% glycerol solution and then frozen in -20 °C for further assessment.

Step 2nd: the determination of the IgG, IgM and IgA antibody activity against the L2 and L3 antigens with the self-developed ELISA and then the determination of the activity of these antibodies in the serum collected from horses immediately after slaughter.

Step 3rd: the IgG, IgM and IgA antibody reactivity against *G. intestinalis* was determined in the saliva of 79 horses with infection intensity determined post slaughter.

### Material

All animal’s tissue have been collected post-mortem, immediately after the slaughter in the slaughterhouse (does not require the approval of the ethics committee). All welfare guidelines complied with national legislation. All methods applied in the study were performed in accordance with the ARRIVE guidelines and regulations. The material for respective study stepa was collected once per month for 1 year. Between January and December 2006 in the horse slaughterhouse a post-slaughter assessment was performed in 513 horses of various sexes, whose average age was 4 years. Among the assessed horses, 263 individuals were uninfected and 250 were infected (which includes 216 individuals in whose cases solely *G. intestinalis* larvae were found). Finally, material from 125 infected horses and 54 uninfected horses were used for further analyzes, which was caused by limitations in obtaining complete material or equivocal results of macroscopic examination.

For 1st step of the study larvae were collected from stomach and duodenum from 10 individuals of infected horses. In 2nd step of the study, blood was obtained by a jugular vein puncture from 90 individuals immediately after slaughter and then, after the inspection of the gastrointestinal tract. Sixty individuals were selected from this group: 30 horses suggestive of a natural infection which was confirmed by the presence of at least 50 larvae in the stomach and duodenum after the slaughter and also 30 horses in whom no larvae were found in in the stomach, duodenum and rectum (the horses where the number of larvae was > 1 and < 50 were disqualified from further assessment). In step 3rd, saliva was collected from 110 horses after slaughter (separate individuals than in step 2nd). From this group, on the basis of post-slaughter assessment of the gastrointestinal tract, 55 infected individuals and 24 individuals free from infection were selected for assessment. The horses were divided into 4 groups depending on the number of larvae in the stomach: I—without infection (24 horses); II—low level of infection: 10–50 larvae in stomach (15 horses); III—moderate level of infection: 51–150 larvae in stomach (20 horses); IV—severe level of infection – > 200 larvae in stomach (20 horses). Saliva was collected from horses with the use of Salivette [Salivette (cotton swab w/o preparation), Starstedt, 51.1534] set^25^. On account of the work system in the slaughterhouse, it was impossible to collect, at the same time, saliva and blood from the horse whose gastrointestinal tract was then evaluated macroscopically. After the assessment of the segments of the gastrointestinal tract of the horses for the presence of botfly larvae, the animals were divided into infected and uninfected individuals (in step 2nd) and also to four specific groups (in step 3rd). No information was available about the medical history of the horses, the type of feeding or anti-parasitic treatment.

## Methods

### Antigen isolation

In order to perform the evaluation of the humoral immunological response of the horses, the antigen of *G. intestinalis* was isolated from L2 and L3, defined on the basis of the larvae characteristics.^23,24^ On account of the fact that the botfly larvae are in contact with the mucosal membranes of the host with their mouthpart, it was decided to isolate a mixture of antigens coming both from the anterior part of the larvae body, i.e. the pseudocefalon and the first three trunk fragments and also the entire larvae. In the case of L2, the anterior body part was isolated from 3 larvae (the pseudocefalon, the first three trunk fragments) with a weight of about 80 mg and 1 whole larva was isolated separately (weight: 60 mg). In the case of L3, the anterior part of the body was collected from 3 larvae weighing 300 mg and 1 whole larva was isolated separately (weight: 420 mg). The isolation of the antigen was carried out on the basis of the described methods^10,11^. In order to isolate the antigen, the larvae were frozen in liquid nitrogen and then homogenized in a metal mortar. Then 4 ml 10% sodium dodecyl sulphate (SDS) was poured over the suspensions created in this way. Then the mixture was incubated for one hour at room temperature, while being mixed and then the material was centrifuged with 5300*g* for 15 min. Supernatants were then filtered through bacteriological filter papers with a pore diameter of 0.20 μm, and then diluted in PBS buffer pH 7.4. The buffer was changed three times, every three hours. After the dialysis, the protein concentration was measured with colorimetiric method (Bicinchoninic Acid Kit for Protein Determination; Sigma, BCA-1) and then the sample was frozen in − 20 °C for further experiments.

### Protein electrophoresis in polyacrylamide gel with SDS (SDS-PAGE)

The separation of protein fractions was performed in the Mini Protean II chamber (Mini Protean II chamber; Bio-Rad) with the use of 10% polyacrylamide gel. Protein samples [5 μg] were placed with a buffer, at a ratio: 1:2. Then mixture was denaturized thermally for 5 min at 95 °C, and then centrifuged 1000*g* for 10 min in room temperature and subsequently placed onto the gel. The electrophoretic separation was performed with a constant current of 20 mA per plate, till the moment when the dye front started to get into the separating gel; then the separation was continued with a current of 30 mA per plate. The separation was stopped once the dye front left the gel. After the electrophoresis, the gel was stained for 12 h in the Coomassie blue 0.1% (Coomassie blue 0.1% R250; Sigma-Aldrich). After staining, the gel was placed on a wash solution consisting of 40% methanol and 10% acetic acid on the laboratory shaker until the moment when the background was removed.

### Detecting antibodies against the obtained *G. intestinalis* antigens in blood serum and saliva using ELISA

The ELISA was performed in two variations on flat-bottom ELISA microplates (Nunc MaxiSorp flat-bottom; Invitrogen, 44-2404-21) with a well volume of 350 ul In the case of serum, the plates were coated with a mixture of antigens isolated from the L2 and L3. In the case of the saliva analysis, the plates were coated with a mixture of antigens isolated only from the L3 (due to the limited volume of saliva).

The formerly extracted *G. intestinalis* antigen at a concentration of 10 μg/ml in 0.05 M bicarbonate buffer pH 9.6 was poured (50 μl/well) and then incubated at a temperature of 37 °C for 3 h. Wells coated with bovine albumin (1 mg/ml of 0.05 M bicarbonate buffer system with pH 9.6) were used as the negative control. Then the plates were incubated overnight at 4 °C. Then 50 μl serums diluted 1:100 in PBS buffer with 0.05% Tween, pH 7.4 (TPBS). TPBS or 50 μl saliva samples diluted 1:10 in TPBS, were added to the wells, with two-fold repetitions for each one sample. Then the microplates were incubated for 2 h on the laboratory shaker 50 RPM at room temperature. For the detection of specific anti-*G. intestinalis* antibodies of IgG, IgM and IgA class in serum and in saliva, conjugated anti-horse IgG (Goat Anti-Horse IgG Antibody, HRP Conjugated; Novus Biologicals), anti-horse IgM (Goat Anti-Horse IgM Antibody, HRP Conjugated; Novus Biologicals) and anti-horse IgA (Goat Anti-Equine IgA Antibody, HRP Conjugated; AbD Serotec) antibodies were used as detection antibodies (diluted 1:20,000 with TPBS).The incubation was carried out on the laboratory shaker for 90 min at room temperature. After the steps of the coating and blocking, first and second antibody, the plates were rinsed three times with 150 μl TPBS buffer with the use of a multichannel pipette. An enzymatic reaction was induced by placing 100 μl of the substrate solution (5 mg o-phenylenediamine in 10 ml 0.05 M of citrate–phosphate buffer, pH 5.0 and 3 μl H_2_O_2_). The incubation was carried out at room temperature in a dark for 15 min. The reaction was blocked by adding 25 μl 2 M of sulphuric acid. The absorbance values were read (Spektra II reader; SLT Labinstruments, Austria) with a wavelength of 492 nm.

### Statistical analysis

Data presented in the tables are means (± SD) (standard deviation). The threshold of statistical significance was set at *p* ≤ 0.05. Differences in reactivity of IgG, IgM and IgA anti-L2 and L3 antibodies between healthy and infected horses were tested using Student's t-test. One-way ANOVA and Tukey Unequal N HSD as post hoc analysis, were used to evaluate the effect of the intensity of *G. intestinalis* infection on the reactivity of antibodies in horses.

## Supplementary Information

Supplementary Figure 1.

## Data Availability

The data that support the findings of this study are available from the corresponding author upon reasonable request.

## References

[1] Cogley TP, Cogley MC (2000). Field observations of the host-parasite relationship associated with the common horse bot fly, *Gasterophilus intestinalis*. Vet. Parasitol..

[2] Niedźwiedź A, Borowicz H, Nicpoń JM (2013). Prevalence study in horses infected by *Gasterophilus* sp. in an eastern region of Poland. Vet. Parasitol..

[3] Gökçen A, Sevgili M, Altaş MG, Camkerten I (2008). Presence of *Gasterophilus* species in Arabian horses in Sanliurfa region. Turk. Parazitol. Derg..

[4] Edens LM, Murray MJ (1992). Gastro-oesophageal reflux in a weanling filly: Association with *Gasterophilus* spp. infestation. Equine Vet. J..

[5] Griss R, Simhofer H (2006). First-time endoscopic detection of larvae of *Gasterophilus* spp. in the oral cavity in 14 warmblood horses. Berl. Munch. Tierarztl. Wochenschr..

[6] Rezazadeh F, Gharehaghajlou Y (2020). Endoscopic finding of gastric ulcer in rural horse and relation with *Gasterophilus* spp. Iran. J. Vet. Med..

[7] Cogley TP (1989). Effects of migrating *Gasterophilus intestinalis* larvae (Diptera: Gasterophilidae) on the mouth of the horse. Vet. Parasitol..

[8] Reinemeyer CR, Scholl PJ, Andrews FM, Rock DW (2000). Efficacy of moxidectin equine oral gel against endoscopically-confirmed *Gasterophilus nasalis* and *Gasterophilus intestinalis* (Diptera: Oestridae) infections in horses. Vet. Parasitol..

[9] Sánchez-Andrade R, Cortiñasa FJ, Franciscoa I, Sáncheza JA, Mulab P, Cazapala C, Vázqueza L, Suáreza JL, Franciscoa R, Ariasa MS, Díez-Bañosa P, Scalab A, Paz-Silva A (2010). A novel second instar *Gasterophilus* excretory/secretory antigen-based ELISA for the diagnosis of gasterophilosis in grazing horses. Vet. Parasitol..

[10] Escartin-Peña M, Bautista-Garfias CR (1993). Comparison of five tests for serologic diagnosis of myiasis by *Gasterophilus* spp. larvae (Diptera: Gasterophilidae) in horses and donkeys: A preliminary study. Med. Vet. Entomol..

[11] Ziomko I, Cencek T (2001). Opracowanie zestawu diagnostycznego do wykrywania przeciwciał anty-Hypoderma bovis u bydła testem Elisa. I. Uzyskanie komponentów. Wiad. Parazyt..

[12] Martinez Moreno FJ, Wassall DA, Becerra Martell C, Hernandez Rodriguez S (1994). Comparsion of the use of secretory and somatic antigens in an ELISA for the serodiagnosis of hypodermosis. Vet. Parasitol..

[13] Innocenti L, Masetti M, Macchioni G, Giorgi F (1995). Larval salivary gland proteins of the sheep nasal bot fly, (*Oestrus ovis* L.), are major immunogens in infested sheep. Vet. Parasitol..

[14] Tabouret G, Prevot F, Bergeaud JP, Dorchies P, Jacquiet P (2001). *Oestrus ovis* (Diptera: Oestridae): Sheep humoral immune response to purified excreted/secreted salivary gland 28 kDa antigen complex from second and third larvae. Vet. Parasitol..

[15] Sánchez-Andrade R, Romero JA, Suárez JL, Pedreira J, Díaz P, Arias M, Paz-Silva A, Panadero R, Díez-Baños P, Morrondo P, Scala A (2005). Comparison of *Oestrus ovis* metabolic and somatic antigens for the immunodiagnosis of the zoonotic myiasis oestrosis by immunoenzymatic probes. Immunol. Investig..

[16] Roelfstra L, Deeg CA, Hauck SM, Buse C, Membrez M, Betschart B, Pfister K (2009). Protein expression profile of *Gasterophilus intestinalis* larvae causing horse gastric myiasis and characterization of horse immune reaction. Parasit. Vectors.

[17] Viviers PL, Elazhary MASY, Lagacè A, Roy RS, Tremblay A (1974). Une étude expérimentale sur la variation du taux danticorps sériques le cycle évolutif des oestres du cheval. Can. J. Comp. Med..

[18] Suárez JL, Scala A, Romero JA, Paz-Silva A, Pedreira J, Arias M, Diaz P, Morrondo P, Diez-Banos P, Sánchez-Andrade R (2005). Analysis of the humoral immune response to *Oestrus ovis* in ovine. Vet. Parasitol..

[19] Lamm ME (1988). The IgA mucosal immune system. Am. J. Kidney Dis..

[20] Aboshady HM, Stear MJ, Johansson A, Jonas E, Bambou JC (2020). Immunoglobulins as biomarkers for gastrointestinal nematodes resistance in small ruminants: A systematic review. Sci. Rep.-Uk.

[21] Rivera-Aguilar V, Hernández-Martinez D, Rojas-Hernández S, Oliver-Aguillón G, Tsutsumi V, Herrera-González N, Campos-Rodriguez R (2000). Immunoblot analysis of IgA antibodies to *Naegleria fowleri* in human saliva and serum. Parasitol. Res..

[22] Lightbody KL, Davis PJ, Austin CJ (2016). Validation of a novel saliva-based ELISA test for diagnosing tapeworm burden in horses. Vet. Clin. Pathol..

[23] Draber-Monko A (1978). Gzy (Diptera: Gsterophilidae, Hypodermatidae i Oestridae) pasożyty ssaków Polski. Monogr.

[24] Zumpt F (1965). Myiasis in Man and Animals in the Old World.

[25] Linder A, Marx S, Kissenbeck S, Mosen H (2000). Saliva collection and relationship between lactate concentration in blood and saliva of exercising horses. J. Equine Vet. Sci..

