# Supplementary figures and images for: The relationship between the intensity of Gasterophilus intestinalis larvae infection and the serum and salivary humoral immune response in horses
Source: Sci Rep. 2022 Oct 20;12:17573. doi: 10.1038/s41598-022-21482-z (PMC9585061; doi:10.1038/s41598-022-21482-z)

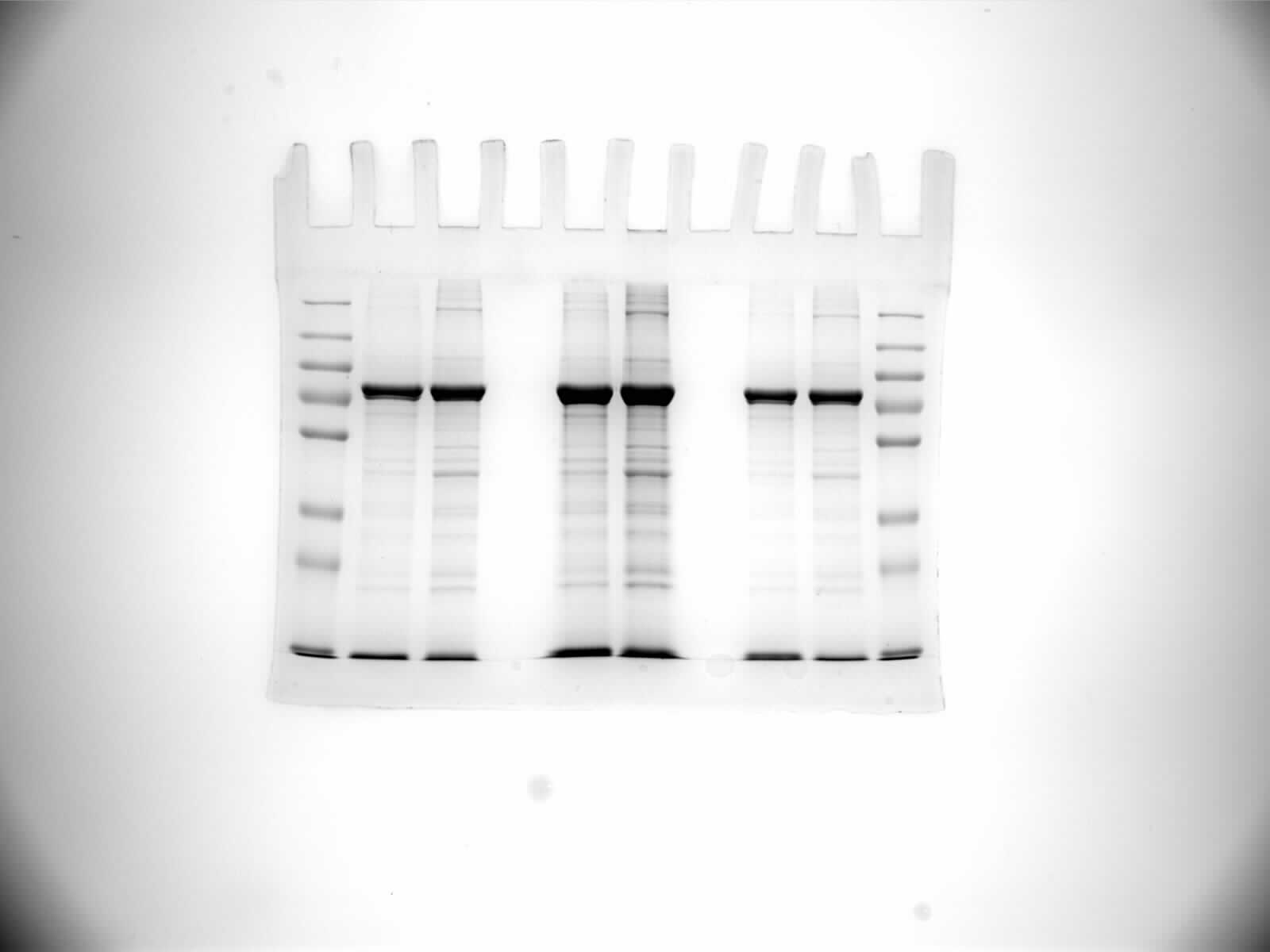

Supplement: Supplementary file 1 — Supplementary Figure 1. [file 41598_2022_21482_MOESM1_ESM.jpg]
